# The Usage and Effects of Restrictive Practices on Patients With Anorexia Nervosa in Paediatric Mental Health Hospitals in the UK

**DOI:** 10.1192/bjo.2026.11612

**Published:** 2026-06-30

**Authors:** Phoebe O'Connor

**Affiliations:** University Of Manchester, Manchester, United Kingdom

## Abstract

**Aims:**

There is an oxymoronic state of our country's paediatric inpatient units treating anorexia nervosa, a deadly illness deeply rooted in restriction and control. These hospitals, which are meant to be places of safety and support, can be some of the most restrictive and unsafe environments patients have found themselves in, being stripped of too many things that make us feel human.

The emergence nationally known cases such as the death of Ruth Szymankiewicz in 2022, who was being looked after in an NHS paediatric inpatient unit for anorexia nervosa, have highlighted gaps in the mental health system that threaten patient safety. However, more needs to be done to protect our most vulnerable, and making a national inter-disciplinary effort to review inpatient hospital policies and procedures for treating anorexia nervosa is an essential step in making that change.

The aim of this report is not to blame people or institutions, but to highlight accounts of systematic failure faced by our paediatric patients that have been ignored for too long and that could have been prevented.

**Methods:**

I composed an online questionnaire, firstly outlining eligibility, then explaining the purpose of the questionnaire and the potential uses of the data they share, then gaining consent to share the information in my report, before proceeding to the questions.

They were then invited through a series of questions to reflect on how they felt they were treated during their inpatient stay and how they feel their experiences impacted them. Due to the age and sensitivity of the topic, the questionnaires were filled in anonymously, and any other identifiable information has been omitted.

**Results:**

Even acknowledging the impact illness can have on memory, there are still several harrowing accounts shared in the report of excessive abuse, infringement on safety, including toilet supervision of female minor patients by male staff, and physical and mental harm, including accounts of patients being refused treatment and the ability to see family, all not conducive to recovery.

**Conclusion:**

Whilst the NHS prides itself on equality, these accounts reveal the varying levels of care and negligence received in units where patients as young as 8 can be admitted. Some recall outstanding support from staff and their hospital environment, and I believe it is our duty as healthcare professionals to ensure that every child battling anorexia nervosa has their needs and rights to feel safe and valued, not endangered met.

